# A technique for the management of posttraumatic aniridia and aphakia

**DOI:** 10.3205/oc000146

**Published:** 2020-04-02

**Authors:** Rita Sousa Silva, Carolina Pereira Bruxelas, Gabriel Costa Andrade, André Correa Maia

**Affiliations:** 1 Department of Ophthalmology, Instituto de Oftalmologia Dr. Gama Pinto, Lisbon, Portugal; 2 Department of Ophthalmology, Hospital Egas Moniz, Lisbon, Portugal; 3 Department of Ophthalmology, Universidade Federal de São Paulo, São Paulo, Brazil

**Keywords:** aniridia, aphakia, iris prosthesis, trauma

## Abstract

**Aim:** To describe our results with HumanOptics IOL-Artificial*Iris* complex in post traumatic aphakia and aniridia.

**Methods:** Retrospective, single-surgeon chart review of cases in which aniridia and aphakia were corrected using HumanOptics IOL-Artificial*Iris* complex sutured to the sclera with Gore-Tex^®^ sutures and coupled with the Akreos^®^ IOL (Bausch&Lomb).

**Results:** The authors present four cases of ocular trauma with globe rupture. For every patient, posterior vitrectomy was done and an artificial iris-lens diaphragm was sutured to the sclera. All patients had a good functional and cosmetic outcome.

**Conclusions:** Surgical implantation of the HumanOptics IOL-Artificial*Iris* complex coupled with the Akreos^®^ IOL was successful in alleviating post-trauma aphakia and aniridia related visual impairment.

## Introduction

With its ability to control the amount of light that reaches the retina through pupillary diameter changes, the iris has a paramount role in vision quality. Some conditions, either congenital or acquired, can contribute to complete or partial iris absence with serious consequences to vision. These include diminished visual acuity, spherical aberrations, glare, photophobia, contrast perception and changes in depth of focus. There is also an important cosmetic factor [1], [2], [3]. Nowadays there are several surgical and non-surgical options to correct iris defects, such as cosmetic contact lenses, corneal tattooing, intrastromal corneal implants, microtying suture techniques, amongst others. For defects so large that they are impossible to correct surgically, there is the option of prosthetic iris implants [1].

Used for the first time in 1964 by Peter Choyce, iris prostheses have undergone major changes. Presently, there are several models available, such as IOL-iris combinations, capsular tension ring based prosthetic iris devices and foldable custom artificial iris [1]. As far as the IOL-iris prostheses combinations are concerned, the first models consisted of a central optic, a 10 mm PMMA diaphragm with a black outer ring, curved haptics, and fixation loops [2]. There are now more modern devices such as the Morcher iris IOL implants and the Ophtec BV. The first offers correction for both aniridia and aphakia, however it requires a large corneal incision (150–180º) and the iris is pure black which cosmetically is not ideal. As for the second device, although it comes in three colours, it also requires a large incision and still has a poor disguised artificial appearance when compared to the fellow eye.

In 2007, the HumanOptics Custom*Flex*^®^ Artificial*Iris* prosthesis became available. It consists of a foldable custom made hydrophobic silicone diaphragm, with the option of an integrated polymer fiber meshwork to which an IOL can be sutured to correct both aniridia and aphakia [1], [2], [3]. The authors present a retrospective, single-surgeon chart review of four cases in which aniridia and aphakia were corrected with an IOL-artificial iris complex sutured to the sclera using Gore-Tex^®^ sutures. All patients underwent complete ophthalmic examination both before and after the procedure.

## Case descriptions

**Figure 1:**
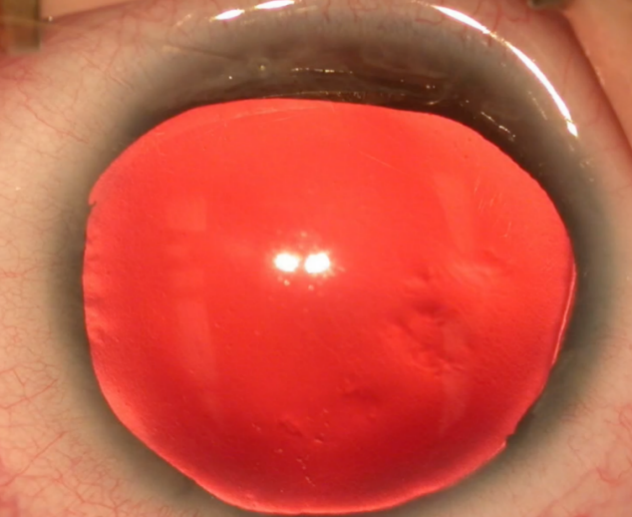
Anterior segment photograph of case 1, showing almost complete lack of iris tissue

All patients included in this review (Table 1 (Tab. 1)) had suffered ocular trauma with globe rupture, iris disinsertion, and traumatic cataract (Figure 1 (Fig. 1)). Mean age was 54.5 years (min. 45, max. 63), with three men and one woman included in this case series. There were no known previous ophthalmological problems. Visual acuity in the preoperative period was hand motion or less in all patients.

**Figure 2:**
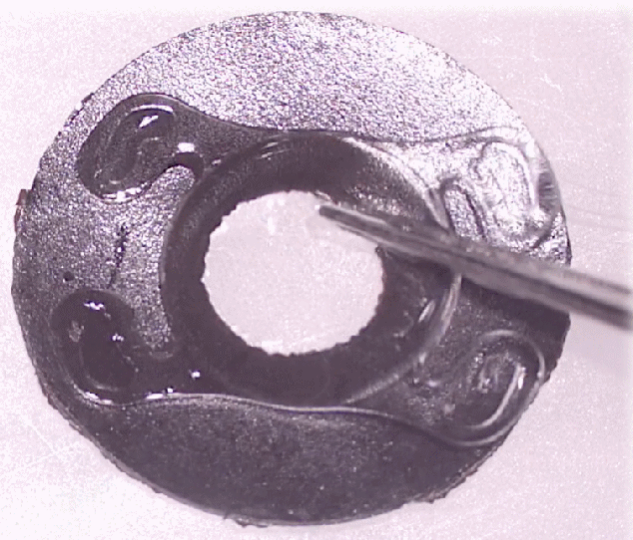
The Akreos^®^ IOL-Artificial*Iris* complex

**Figure 3:**
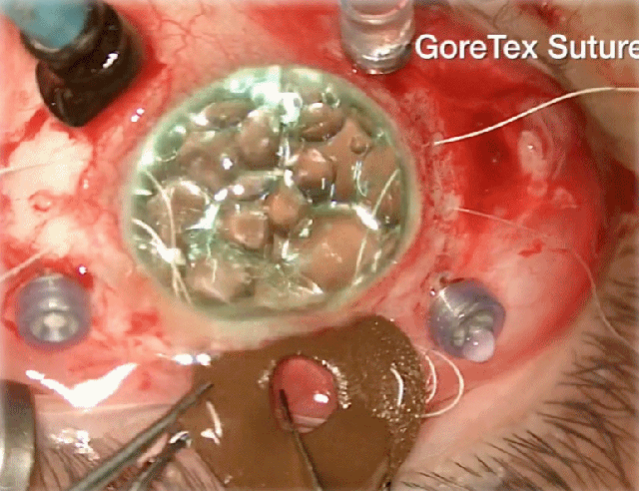
Surgical procedure with implantation of the Akreos^®^ IOL-Artificial*Iris* complex

25-gauge pars plana vitrectomy was done in all patients. Patient 2 also had phacoemulsification done for partial spontaneous cataract resorption. In all cases, a previously measured and cut Custom*Flex*^®^ Artificial*Iris* prosthesis (Human Optics) and an Akreos^®^ IOL (Bausch&Lomb) was folded and dialed into place in the ciliary sulcus, through the superior 3.75 mm limbal-based corneal wound. The IOL was sutured to the mesh on the posterior face of the artificial iris with 10-0 polypropylene sutures, first anteriorly to posteriorly and then posteriorly to anteriorly through the IOL eyelets (Figure 2 (Fig. 2), Figure 3 (Fig. 3)). 

The Akreos^®^ IOL-Artificial*Iri*s complex was fixed and centered to que sclera, using two equidistant 7-0 CV-8 Gore-Tex^®^ sutures. The sutures were trimmed, rotated, and buried, only covered by conjunctiva.

The corneal wound was then closed using a 10-0 nylon suture [3], [4], [5], [6]. 

## Discussion

Trauma to the eye with subsequent aniridia and aphakia not only has functional consequences with poor quality of vision, but also the aesthetic component can be a constant reminder of a traumatic event [1], [2]. 

The authors present a refined approach to this condition, using a custom-made artificial iris, to match the patient’s original iris color, and to which an adequate IOL was sutured. The flexible and foldable artificial iris has a diameter of 12.8 mm and can be easily cut with scissors or with a trephine and then inserted through small incisions into the ciliary sulcus of a pseudophakic or aphakic eye [1], [3]. 

**Figure 4:**
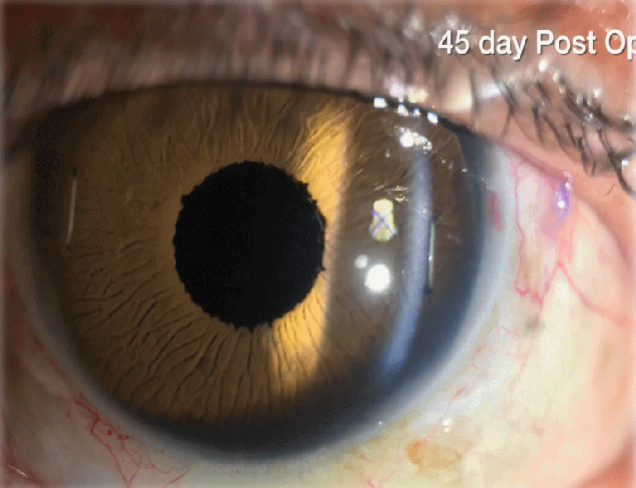
Postoperative appearance at the slit-lamp

The results showed not only a remarkable aesthetic result (Figure 4 (Fig. 4)) but also a significant improvement in visual acuity, like in other series [3]. Six months after surgery, BCVA was between 20/150 (patient 1) and 20/50 (patient 4). Vitreous hemorrhage and hyphema were intraoperative complications in patients 2 and 3, respectively. Secondary glaucoma was the only late postoperative complication and it happened in one patient (patient 2). No evidence of suture erosion or breakage, wound leak or infectious endophthalmitis was observed in either case. 

Despite these very positive results, one of the four cases reviewed developed late glaucoma with requiring ongoing medication after surgery. Some authors associate late complications such as the latter as well as darkening of the iris tissue and the need for consecutive anterior segment surgery, with the presence of the integrated polymer fiber meshwork present in these artificial iris, presumably due to their sharp endings [3]. 

## Conclusions

In trauma-related aphakia and partial or complete iris defect, surgical implantation of the HumanOptics IOL-Artificial*Iris* complex coupled with the Akreos^®^ IOL appears to be a satisfactory method for improvement of both visual function and cosmetic issues. 

However, further experience and longer follow-up times are needed in order to best determine the true incidence of long term complications with this method and how to avoid them [1], [3].

## Notes

### Conference presentation

Best poster presentation at the 3^rd^ European Meeting of Young Ophthalmologists, July 13-14, 2018 in Cracow, Poland.

### Competing interests

The authors declare that they have no competing interests.

## Figures and Tables

**Table 1:**
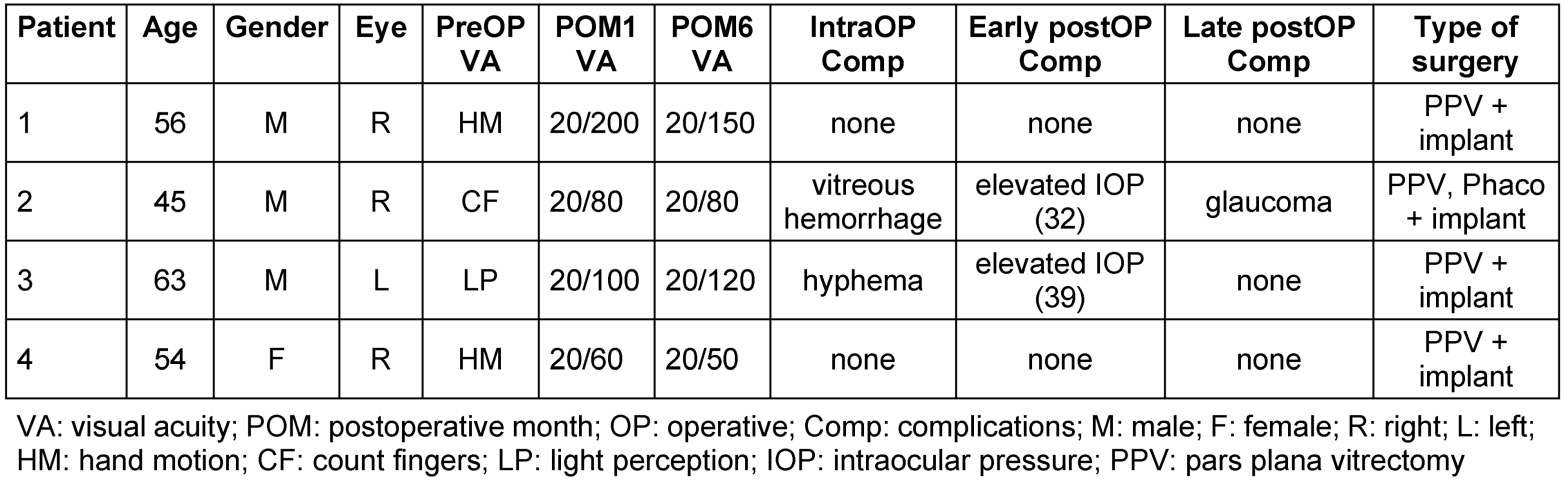
Patients with aniridia and aphakia corrected with IOL artificial iris complex
